# Ultrasound and radiographic outcomes of the Coxaflex brace in the treatment of developmental dysplasia of the hip (DDH): a multicentre prospective study

**DOI:** 10.1007/s00590-026-04942-4

**Published:** 2026-09-10

**Authors:** Maria Rizzo, Michela Saracco, Clara De Negri, Valerio Verdoliva, Francesco Maria Lotito, Antonio Colella, Maria Paola Miolla, Daniela Dibello, Massimo Mariconda

**Affiliations:** 1 Department of Public Health - Section of Orthopaedics and Trauma Surgery, Federico II University, Naples, Italy; 2 Unit of Pediatric Orthopaedics and Traumatology, Giovanni XXIII Pediatric Hospital, Bari, Italy

**Keywords:** Developmental dysplasia of the hip, Coxaflex brace, Dynamic brace, Infant hip instability

## Abstract

**Purpose:**

Early diagnosis is essential for the treatment of developmental dysplasia of the hip (DDH), and the use of abduction bracing is considered the gold standard for patients < 6 months of age. The aim of this study is to report an analysis of the results of using the Coxaflex abduction brace.

**Methods:**

This multicentre prospective study included 44 hips in infants younger than 6 months with unilateral or bilateral reducible (Barlow-positive) developmental dysplasia of the hip, treated with the Coxaflex brace. DDH was diagnosed by clinical examination and ultrasound during outpatient screening. Serial ultrasound examinations were performed throughout treatment, while pelvic radiographs were obtained at the end of treatment to assess the acetabular index (AI) and the appearance of the proximal femoral ossification nucleus.

**Results:**

The mean age at the start of treatment was 46 days (range, 4–150 days). The first follow-up (T1) was performed at 42 ± 13 days of treatment, a second follow-up (T2) at 91 ± 21 days, and a third follow-up (T3) at 134 ± 22 days. Ultrasound examinations were performed at each follow-up, which revealed a mean α angle of 47°±4° at the start of treatment. At the T1 follow-up, the mean α angle was 57°±7°, while on T2 and T3 they measured 63°±5° and 64°±5°, respectively. The angular increase on T1 and T2 was statistically significant (*p* < 0.001). The X-ray examination was performed at an average of 162 ± 40 days and showed an AI of 23°±5° (range 14°-38°). Treatment was successful in all 44 hips. Three hips showed residual acetabular dysplasia at the time of ultrasonographic normalization and required prolonged brace treatment; all achieved radiographic normalization during follow-up. No patient required surgical treatment or conversion to another orthosis.

**Conclusions:**

The Coxaflex brace appears to be an effective and well-tolerated treatment option for infants younger than 6 months with reducible (Barlow-positive) DDH. Since fixed or irreducible hip dislocations were not included in this study, these findings should not be generalized to those patients.

**Supplementary Information:**

The online version contains supplementary material available at https://doi.org/10.1007/s00590-026-04942-4.

## Introduction

Developmental dysplasia of the hip (DDH) is a congenital disorder of the hip joint that ranges from mild acetabular dysplasia to complete hip dislocation. It is one of the most common orthopaedic conditions in newborns, with an incidence varying based on factors such as gender, family history, and breech presentation [1].

Early detection and timely treatment are crucial to ensuring proper hip development, preventing long-term complications such as joint instability, gait abnormalities, and early-onset osteoarthritis. Currently, a screening program is implemented to facilitate the early diagnosis of DDH. A significant reduction in the rate of surgery for DDH later in life was shown after the introduction of universal ultrasound screening [2]. Although clinical examination plays a crucial role in the diagnostic process, studies indicate that up to 54% of dislocations have no clinical signs including no Ortolani sign and no risk factors [3]. Nowadays, ultrasound is considered the gold standard in the diagnostic process, this is mainly because the US examination of newborns’ hips allows a morphological study of both the bone and the cartilage components and also permits a dynamic evaluation of hip instability, so a dysplasia can be identified in vivo, immediately in the peri-natal period minimizing exposure to X-ray radiation [4]. The Graf classification is the most widely used system, based on the measurement of the alpha and beta angles, and categorizes dysplastic hips into four main types according to these angular values [5].

The management of DDH depends on the patient’s age and the severity of the condition. Among the most employed therapeutic options are dynamic and static splints, which are selected based on the reducibility of the hip and the developmental stage of the child [6].

Static splints are designed to achieve a **rigid reduction** by immobilizing the hips in a fixed position of flexion and abduction, thereby preventing active joint motion. These devices are typically made of rigid materials. Evidence suggests that, compared with dynamic orthoses, static splints are associated with a higher incidence of complications, particularly avascular necrosis (AVN) [7]. Their most widely accepted indication is in children older than 6–9 months who require prolonged hip abduction because of persistent acetabular dysplasia and/or hip subluxation [8].

In contrast, dynamic splints facilitate dynamic reduction by allowing controlled movement of the lower limbs while maintaining the hips in a position of flexion and abduction. Their primary indication is hip instability that can be managed through concentric reduction in infants younger than 6 months of age. Among the dynamic orthoses used for the treatment of DDH, the Pavlik harness is the most extensively studied and widely adopted device [9]. However, alternative braces, such as the Coxaflex brace, have been developed to provide effective treatment while potentially overcoming some of the limitations associated with other dynamic orthoses.

The Coxaflex brace is designed to achieve stable concentric reduction while allowing controlled hip movement. By maintaining the femoral head concentrically reduced within the acetabulum, it promotes physiological acetabular development and remodelling [6]. Despite its use in clinical practice, evidence regarding its effectiveness remains limited compared with that available for the Pavlik harness. Therefore, the aim of the present study was to evaluate the effectiveness of the Coxaflex brace as a conservative treatment for reducible developmental dysplasia of the hip by analysing its ultrasound and radiographic outcomes, as well as its potential advantages and limitations [10].

Specifically, we assessed ultrasonographic parameters, including changes in the α angle, together with radiographic outcomes, such as the acetabular index (AI), throughout the treatment period. These findings may contribute to refining current treatment protocols and supporting clinical decision-making in paediatric orthopaedics.

## Materials and methods

Data from patients evaluated for DDH between June 2022 and May 2025 were prospectively collected and retrospectively reviewed.

The inclusion criteria were infants younger than 6 months of age with unilateral or bilateral reducible (Barlow-positive) developmental dysplasia of the hip (DDH) [11]. Exclusion criteria included age greater than 6 months at diagnosis, follow-up of less than 6 months, fixed or teratologic hip dislocation (including Graf IV hips), arthrogryposis, stable ultrasonographic hip dysplasia, inability to tolerate the brace because of femoral nerve palsy within the first two weeks of treatment, and poor compliance. Graf IV hips were excluded because the Coxaflex brace is intended for concentrically reducible hips, whereas fixed or irreducible dislocations generally require alternative treatment strategies.

All infants underwent clinical examination by neonatologists at birth, followed by assessment by experienced paediatric orthopaedic surgeons. Infants with positive clinical findings or established risk factors at birth, as well as those without risk factors who underwent routine ultrasonographic screening, were eligible for enrolment. During treatment, patients were reassessed clinically and by ultrasound every 45 days until hip normalization.

Ultrasonographic evaluation included measurement of the Graf alpha and beta angles, as well as documentation of the age at appearance of the proximal femoral ossification nucleus. Hip ultrasound examinations were performed according to the Graf method using a 7.5-MHz linear transducer, allowing accurate in vivo assessment of both hip morphology and stability [5]. All hips were examined sonographically. At the end of treatment, all enrolled infants underwent an anteroposterior pelvic radiograph to assess the acetabular index (AI).

Residual acetabular dysplasia was defined as an acetabular index greater than two standard deviations above the age-specific reference value, according to the criteria described by Tönnis and subsequent revisions [12].

**Fig. 1:**
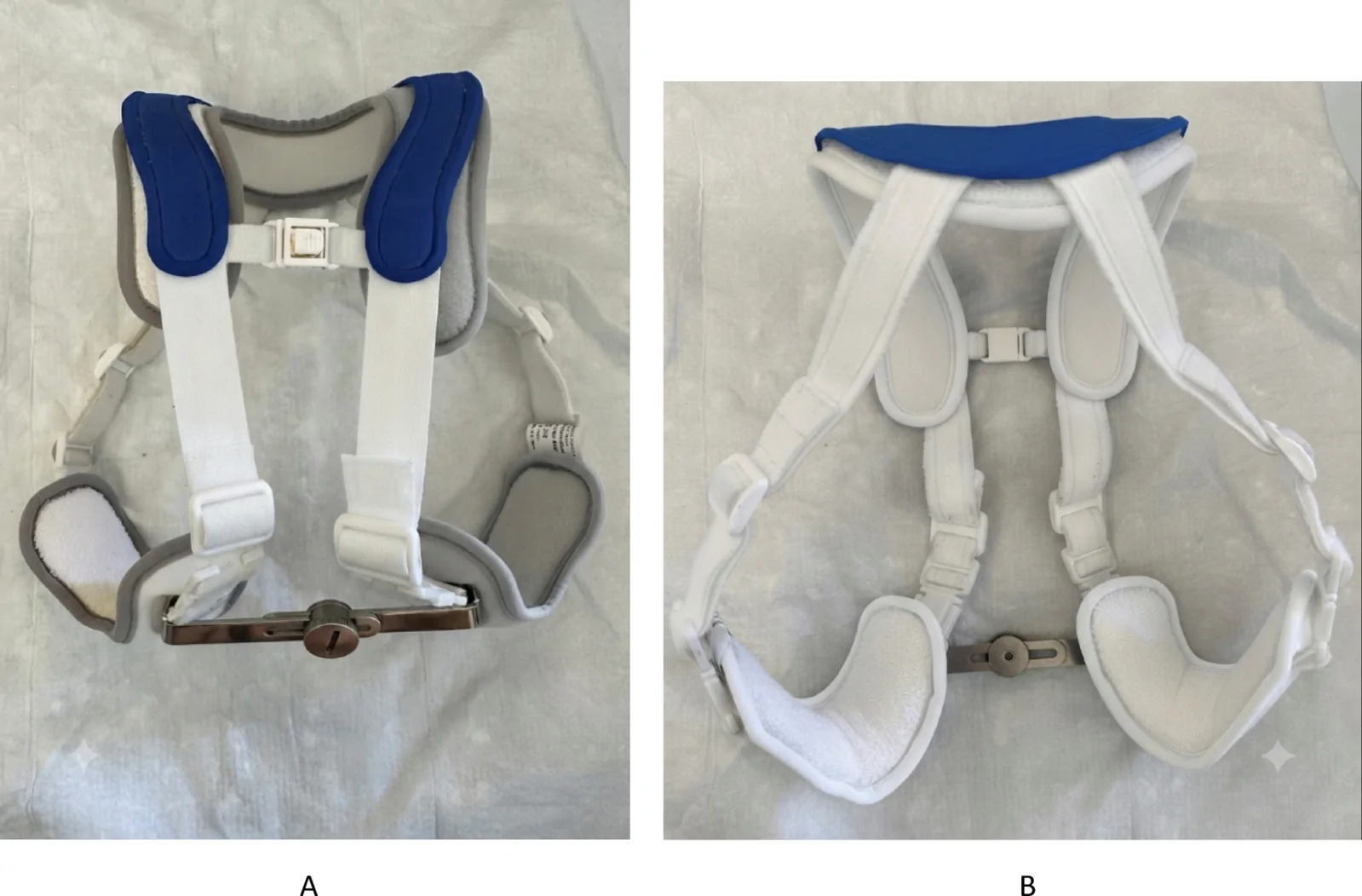

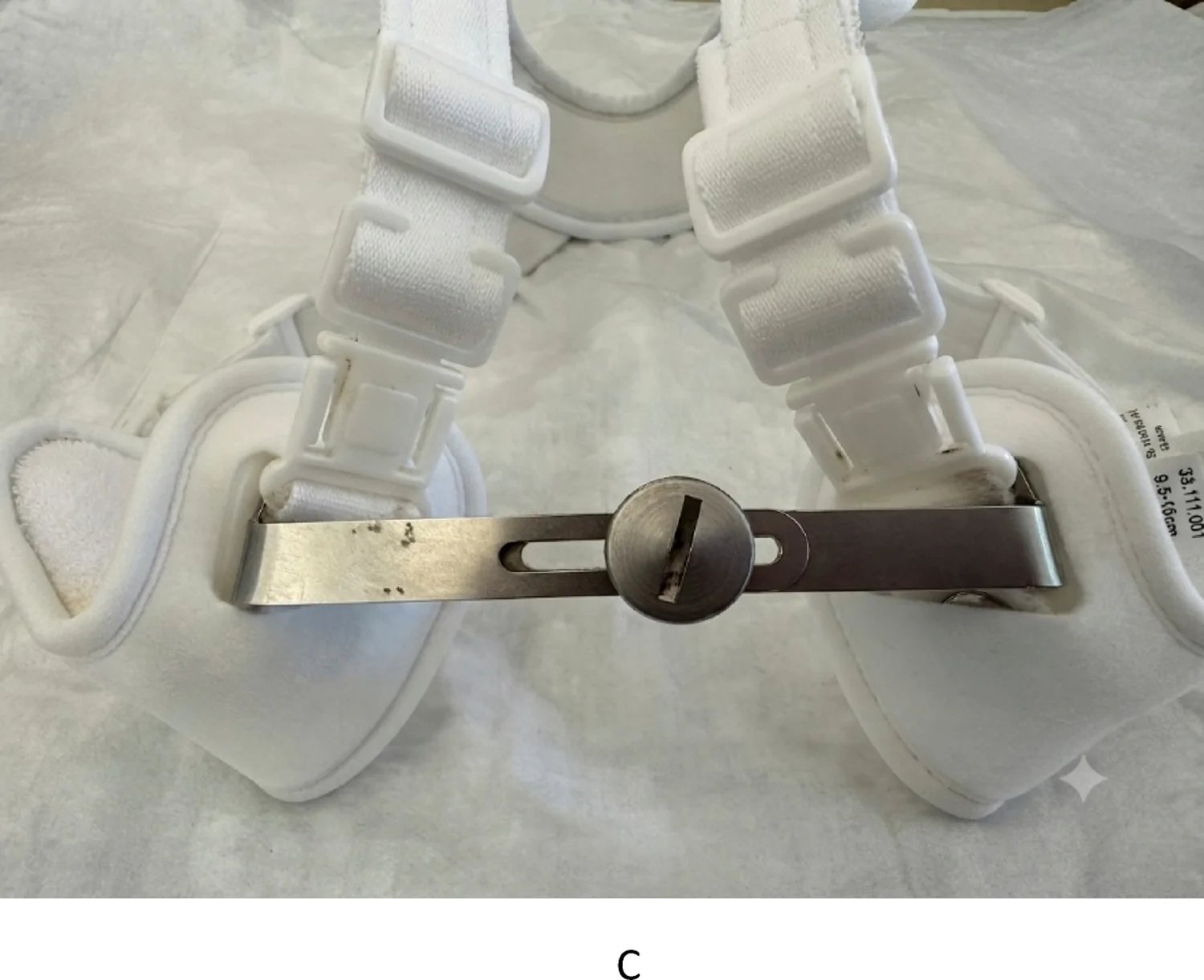
The Coxaflex dynamic hip abduction brace used in this study. **A** Anterior view of the orthosis. **B** Posterior view showing the shoulder support and hip shells. **C** Detail of the adjustable central bar, which allows the clinician to set the desired degree of hip abduction while maintaining hip flexion between approximately 90° and 100° and abduction between 45° and 50°

Treatment consisted of the Coxaflex brace (Fig. 1), a dynamic hip abduction orthosis with greater rigidity than conventional dynamic harnesses. The device is designed to maintain the hips in a controlled position of flexion and abduction, thereby promoting concentric reduction of the femoral head and physiological acetabular development. It incorporates adjustable bars and straps that allow the clinician to individualize the degree of hip flexion and abduction according to the infant’s clinical condition. The orthosis maintains hip flexion between 90° and 100° and hip abduction between 45° and 50°, while allowing limited physiological movement and preventing extreme positions that could compromise comfort or vascular supply. The brace was prescribed for 23 h per day for a minimum of 16 weeks or until ultrasonographic normalization of the hip was achieved.

The following demographic and clinical variables were recorded: sex, laterality, age at diagnosis (days), family history of DDH, associated congenital anomalies, clinical examination findings, Graf classification at the initial ultrasound assessment, serial changes in the alpha and beta angles during follow-up, acetabular index on radiographs, and age at appearance of the proximal femoral ossification nucleus.

Ultrasonographic alpha and beta angles were evaluated at baseline (T0), after approximately 45 days of treatment (T1), and after approximately 90 days (T2). In infants whose alpha angle remained below 60° at T2, an additional ultrasound assessment (T3) was scheduled approximately 45 days later. Before discontinuation of the brace, all infants underwent pelvic radiography. In patients without ultrasonographic or radiographic evidence of proximal femoral ossification at the time of brace removal, ultrasound was repeated three months later.

In patients who achieved ultrasonographic normalization but still had an acetabular index above the 90th percentile, brace treatment was continued with ongoing ultrasonographic monitoring. A repeat pelvic radiograph was obtained at one year of age to reassess the acetabular index.

Ultrasound examinations were performed independently by two experienced paediatric orthopaedic surgeons, and acetabular indices were measured independently by two observers. Whenever discrepancies occurred in the ultrasonographic or radiographic measurements, the images were jointly reviewed until a consensus measurement was reached. Only the consensus value was included in the final analysis.

Clinical, ultrasonographic, and radiographic treatment success was defined as achievement of a stable hip on physical examination together with ultrasonographic normalization, without the need for closed or open reduction or conversion to a rigid abduction orthosis.

Complications, including treatment failure, femoral nerve palsy, and residual acetabular dysplasia, were recorded.

### Statistical analysis

Statistical analysis was performed using SPSS (IBM Statistics). Data was collected using Excel (Microsoft). To incorporate the data of the participants into data analysis regardless of the protocol adherence, all the analyses were performed in accordance with the intention to-treat principle. Appropriate sample size calculation and power analysis were assessed during the planning of the clinical study. Paired t-test or Wilcoxon signed rank test was applied to assess changes after the proposed treatment. Data were expressed as frequencies and percentages for categorical variables and mean and standard deviation (M ± SD) for continuous variables. Intra-class correlation coefficients were used as a measure of concordance in radiographic evaluations between observers. A confidence level of 95% was selected and a *p*-value < 0.05 chosen as significance threshold.

Univariate and multivariate logistic regression analysis was performed to identify predictors of the presence of the proximal femoral ossification nucleus at the end of brace treatment. Age at diagnosis, alpha angle at T0, Beta angle at T0 and treatment duration were tested as possible predictors.

Linear regression analysis was carried out to assess possible predictors of the AI at diagnosis, alpha angle at T0, Beta angle at T0 and treatment duration were tested as possible outcome predictors.

A two-sided *p*-value < 0.05 was considered statistically significant, with a confidence level set at 95%.

## Results

**Fig. 2:**
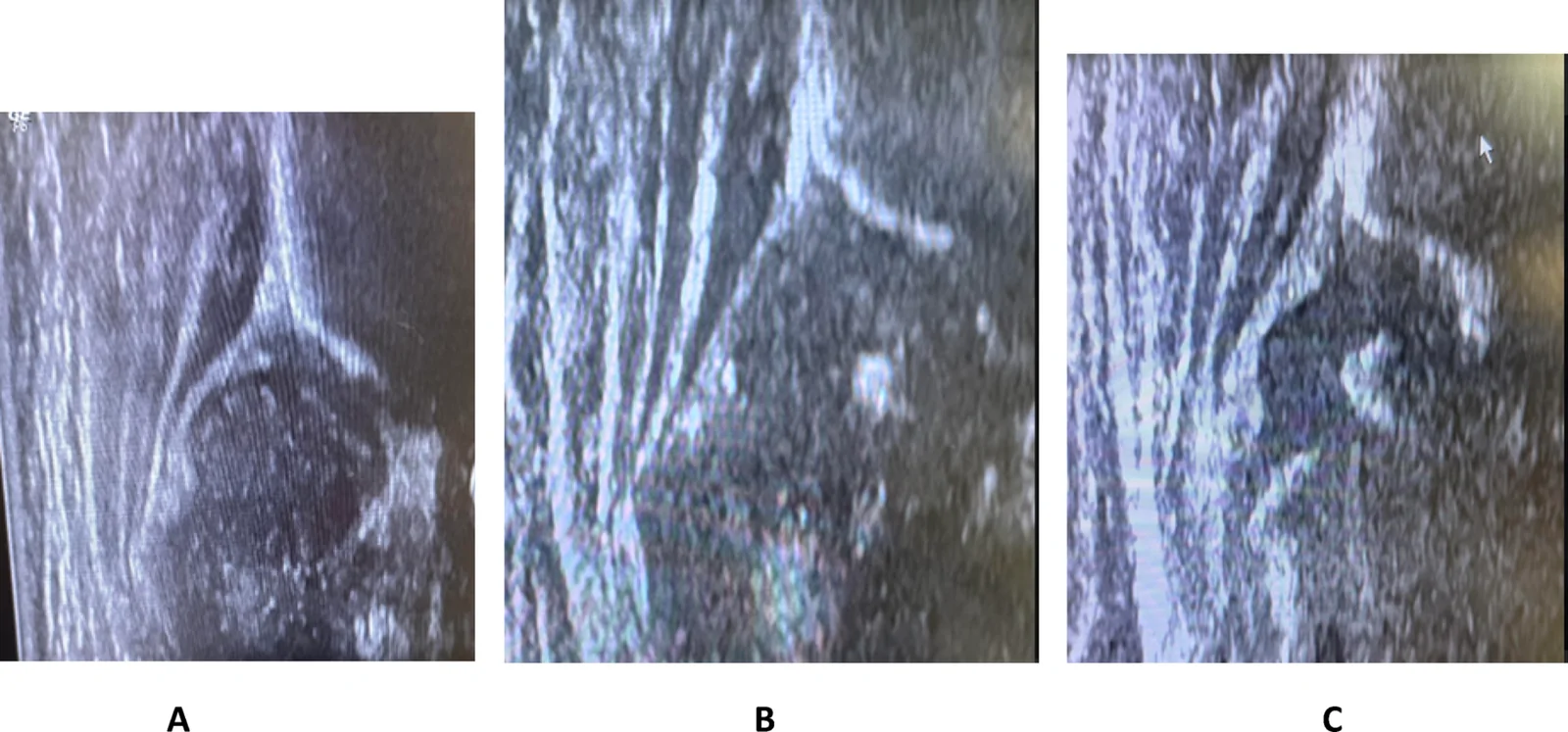

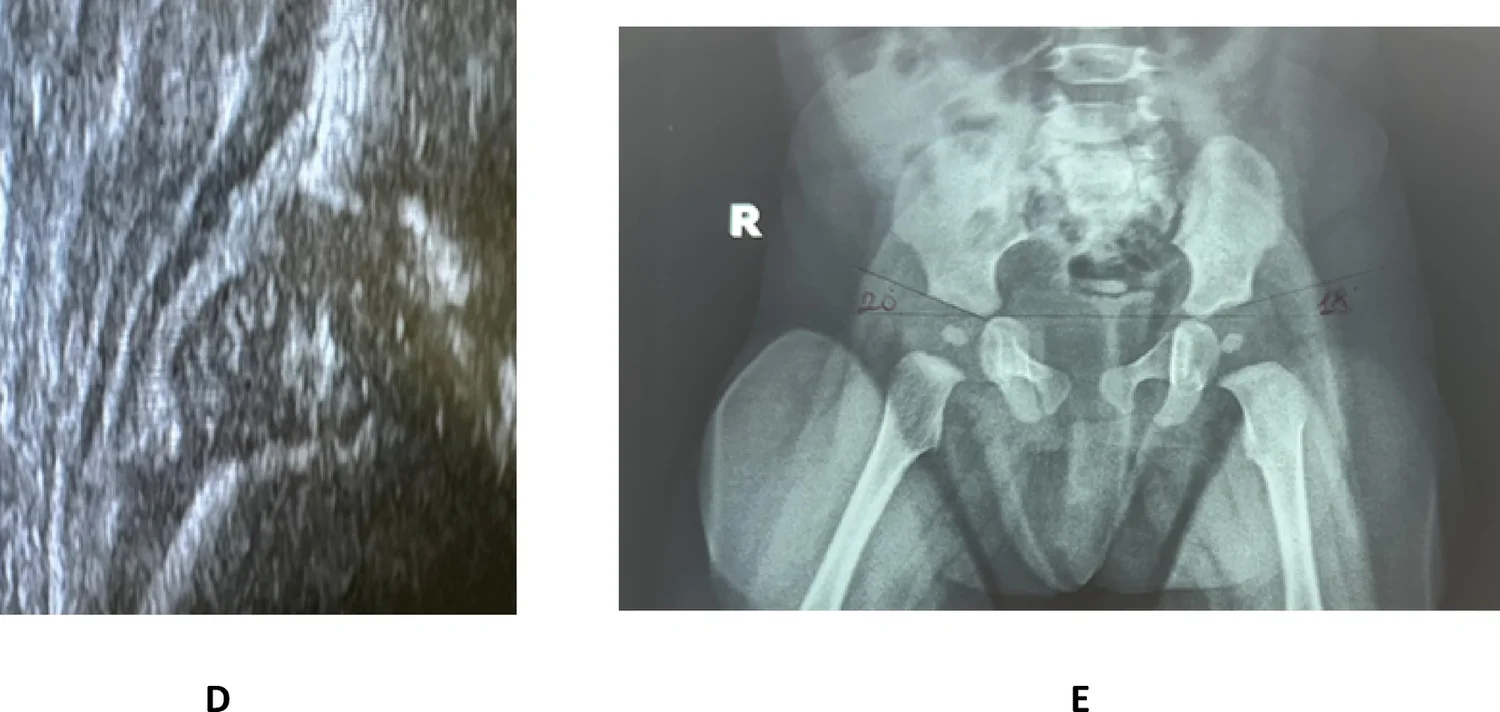
Representative clinical case showing progressive ultrasonographic and radiographic normalization during Coxaflex treatment. **A**: Female, 18 days of age. Born at term, from a spontaneous delivery and with no family history of DDH. Positive Ortolani test on the left side.She therefore underwent hip ultrasonography according to the Graf method, which showed a type 2 C hip on the left with an α angle of 49° and a β angle of 60° (T0). Patient was therefore immediately treated with a Coxaflex brace full-time, with careful parental education regarding its use (worn 23 h/day). **B** 50 days Follow-up (T1): Improvement in the Graf angles, with an α angle of 60° and a β angle of 55°. The ossification nucleus of the femoral head was also visible.** C** Follow-up performed at 90 days (T2) αangle = 67°, while the β angle = 55°.** D** 120 days follow-up (T3) US stabilization of the Graf angle values within the normal range ( α angle = 69° and β = 55°)( Fig. 2**D**). Radiographic follow-up performed at 120 days (Fig. 2**E**). The radiograph showed, on the affected left side, an acetabular index (AI) of 18°, within the normal percentile range for age and sex (< 25°) and comparable to the contralateral side (AI 20°). After normalization of ultrasound and radiographic parameters, the patient discontinued the Coxaflex brace.

A total of 44 hips were evaluated, including 22 unilateral and 11 bilateral cases, comprising 18 right and 26 left hips. The study population included 28 females and 5 males. (Fig. 2)

Detailed demographic data are reported in the **Supplementary Material**.

The median age at diagnosis (T0) was 46 ± 40 days (range, 4–150 days) and the average age at the end of treatment was 224 ± 54 days. The mean alpha angle at T0 was 47 ± 4°, and the mean beta angle was 62 ± 13°. Nineteen hips demonstrated a positive Ortolani manoeuvre. Hip types according to the classification system are summarized in Table 1.

**Table 1:** Distribution of patients and age at the end of treatment according to Graf classification. Data are presented as number of patients (N) and mean age in days ± standard deviation (SD)

Graf Classification	*N* = 44	Age (days) at the end of treatment (M ± SD)
2B	3	300 ± 2
2 C	30	213 ± 49
D	3	254 ± 56
3 A	8	234 ± 66

Interobserver agreement for ultrasound and radiographic measurements was greater than 80%.

Ultrasound follow-up assessments were performed at T1, with a mean interval of 42 ± 13 days; at T2, with a mean interval of 91 ± 21 days; and at T3, where 33 hips were evaluated, with a mean interval of 132 ± 22 days.

**Fig. 3:**
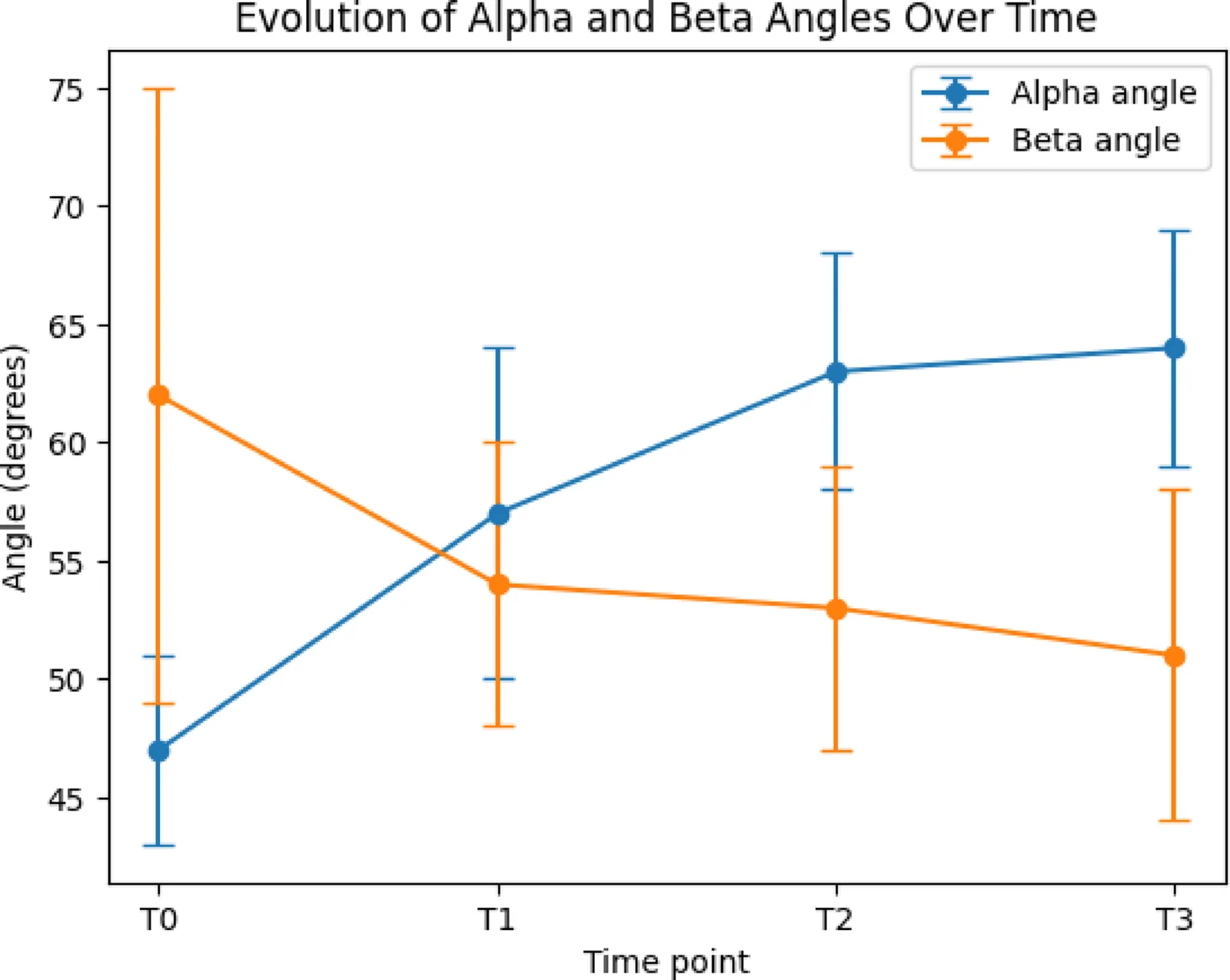
Evolution of alpha and beta angles across the four time points (T0–T3). Data are presented as mean values, with error bars representing standard deviations (SD)

At T1, the mean alpha and beta angles were 57 ± 7° and 54 ± 6°, respectively, showing a statistically significant difference compared with the time of diagnosis (*p* < 0.001). At the second follow-up (T2), the mean alpha and beta angles were 63 ± 5° and 53 ± 6°, respectively. The alpha angle demonstrated a statistically significant increase (*p* < 0.001), whereas the beta angle did not show a statistically significant reduction on paired t-test analysis (*p* = 0.165). (Fig. 3) At the ultrasound evaluation performed at T3, the mean alpha angle was 64 ± 5°, and the mean beta angle was 51 ± 7°, with no statistically significant differences compared with the previous follow-up (*p* = 0.317 and *p* = 0.017, respectively), as shown in Table 2. All ultrasound examinations were performed by paediatric orthopaedic surgeons with expertise in hip ultrasonography. A standard anteroposterior pelvic radiograph was obtained prior to brace discontinuation, at a mean follow-up of 162 ± 40 days. At the time of imaging, patients had a mean age of 208 ± 57 days. This evaluation allowed assessment of the acetabular index (AI) and comparison with age- and sex-specific percentiles. The mean AI was 23 ± 5° (range 14°-38°), consistent with values reported in the literature. At the end of brace treatment, 30 hips (68%) showed ultrasonographic evidence of the proximal femoral ossification nucleus, with a mean age of 164 ± 45 days. However, hips without ultrasonographic or radiographic evidence of the proximal femoral ossification nucleus at the time of brace discontinuation, underwent additional follow-up evaluations, including ultrasound at 3 and 6 months after brace removal and radiographic assessment at 1 year. In all cases, the proximal femoral ossification nucleus was detected at these subsequent post-treatment evaluations.

**Table 2:** Ultrasound assessments at diagnosis and follow-up

Time point	Number of hips	Mean age / interval (days)	Alpha angle (°)	Beta angle (°)	Statistical significance
T0 (Diagnosis)	44	46 ± 40	47 ± 4	62 ± 13	*p* < 0.001
T1	44	42 ± 13	57 ± 7	54 ± 6	
					Alpha: *p* < 0.001; Beta: *p* = 0.165
T2	44	91 ± 21	63 ± 5	53 ± 6	
					Alpha *p* = 0.317; Beta *p* = 0.017
T3	33	132 ± 22	64 ± 5	51 ± 7	

Univariate and multivariate regression analysis was performed to evaluate the association between age at diagnosis and treatment duration with the appearance of the proximal femoral ossification nucleus at the end of brace treatment. Treatment duration was significantly associated with the outcome (B = − 0.024; SE = 0.011; *p* = 0.032; OR = 0.977; 95% CI: 0.956–0.998), indicating that longer treatment duration was associated with a reduced likelihood of ossification nucleus absence at brace removal. Conversely, age at diagnosis was not significantly associated with the outcome (B = − 0.001; SE = 0.008; *p* = 0.942; OR = 0.999; 95% CI: 0.983–1.016).

Furthermore, linear regression analysis demonstrated that age at diagnosis, alpha angle at T0, beta angle at T0 and treatment duration were not significantly associated with the acetabular index and did not represent independent predictors of this radiographic parameter.

Three hips presented an acetabular index above the 90th percentile at the time of ultrasonographic normalization. One female with bilateral dysplasia showed an acetabular index of 33° in the right hip and 38° in the left hip; therefore, a third ultrasound follow-up was performed, and the treatment period was prolonged, resulting in a total duration of 182 days. Another female patient had an acetabular index of 36°; in this case an additional ultrasound follow-up was performed 30 days after the radiographic assessment, with a total treatment duration of 150 days. These patients underwent further radiographic examination at one year of age. The first patient had an AI of 22° on the right and 25° on the left. The second patient had an AI of 22°. In all cases, the growth plate of the proximal femur was present.

Overall, treatment was successful in all 44 hips. Although three hips demonstrated residual acetabular dysplasia at the time of ultrasonographic normalization, all achieved radiographic normalization after prolonged brace treatment. Consequently, no patient remained dysplastic at the latest follow-up, and no patient required surgical intervention or treatment with an alternative orthosis.

No complications were recorded. No infant developed femoral nerve palsy during treatment. Consequently, no patient was excluded for this reason.

## Discussion

The Coxaflex brace is similar to the Pavlik one as a dynamic orthosis but provides greater stability. This makes it easier to use, allowing for improved parental compliance. The Pavlik brace remains the preferred treatment for children under 6 months of age, as it is the most widely described, analysed, and considered safe and highly effective in large samples [13]. Therapeutic success has been reported with the use of other braces, including the Coxaflex (98.3%); however, the small sample size of the study did not allow for a proper comparison with other types of braces [10]. The available literature on this device is still limited especially compared to the Pavlik brace. This disparity was also highlighted by Pavone et al. (2021), who in a systematic review analysed the main static and dynamic braces used in the treatment of DDH [9]. The only study in the literature specifically dedicated to the Coxaflex brace, authored by Azzoni et al. (2011), reported a success rate close to 100% on a sample of 59 patients. The authors also observed that more severe DDH require a longer treatment period and that cases treated late require more time to achieve correction [10]. According to a study by Aarvold et al., the majority of British Society for Children’s Orthopaedic Surgery (BSCOS) members reported that the duration of DDH treatment ranged from 6 to 16 weeks (mostly 12 weeks) [14]. Another survey by Kelley et al. confirmed that most paediatric orthopaedic surgeons believe that DDH treatment should last for three months [15]. Anyway, at the end of the treatment, a control pelvis X-ray is essential to evaluate acetabular angles [16]. 

The systematic review by Merchant et al. found an average duration of full-time brace use of 16.4 weeks for dynamic devices and 8.9 weeks for static ones. Bracing is generally maintained full-time, with one hour per day off for child hygiene [17]. Hines et al. also demonstrated that there are no significant clinical differences between wearing the brace for 23–24 h a day [18]. 

The importance of radiographic normalization, in addition to ultrasound, was reiterated by Li et al. (2022), who in a retrospective study demonstrated the possible persistence of the pathology despite ultrasound normalization upon discontinuation of the brace [19, 20]. Finally, some recent studies support the use of static braces in the treatment of residual acetabular dysplasia in older infants, when the Pavlik brace is no longer indicated [21]. These devices appear to improve the acetabular index, although data are still limited. In many centres, the use of a rigid, part-time abduction brace is common, which can promote acetabular remodelling without significantly interfering with the child’s activity [22]. However, the optimal duration of treatment remains a matter of debate.

The Coxaflex brace showed a low incidence of complications compared to static braces, confirming its conformity with other dynamic devices. Merchant et al. (2021), in his systematic review, emphasized the greater safety and tolerability of dynamic braces compared to static ones, findings later shared by other authors [17, 23]. 

An advantage of static braces is their design, which reduces the risk of parental errors in positioning, allowing for easier application and minimizing the possibility of incorrect use. Furthermore, these devices can also be used in children over six months of age. In the review by Pavone et al., the success rate for static braces was 93%, with a failure rate of 7%. Although the number of cases analysed (212 hips) is relatively limited, the results confirm the effectiveness of this type of treatment [9]. Several authors have also reported cases of stabilization of persistent posterior dislocations after failure of the Pavlik dynamic brace using static ones [21, 24]. 

Zgoda et al. documented that the use of dynamic braces for DDH did not interfere with locomotor development, although it showed an average delay of approximately three weeks in the onset of ambulation, with no long-term functional consequences [25]. 

This study certainly has some limitations: the follow-up is short. It would be interesting to re-evaluate all children at 2 years of age with an antero-posterior pelvic x-ray to exclude the presence of avascular necrosis according to Salter’s criteria. It would also be interesting to expand the sample size. On the other hand, this is the first prospective study on the Coxaflex brace, to the authors’ knowledge.

In conclusion, the treatment of DDH requires a long period of care, not only from the specialist but also from the parents. Therefore, compliance is essential to achieve a successful outcome. The Coxaflex brace is easy to apply and well tolerated by infants, factors that may contribute to successful treatment when combined with early diagnosis. In this prospective cohort, the Coxaflex brace proved to be an effective treatment option for infants younger than 6 months with reducible (Barlow-positive) developmental dysplasia of the hip. Since fixed or irreducible hip dislocations (including Graf IV hips) were not included, these findings should not be extrapolated to those patients.

## Supplementary Information

Below is the link to the electronic supplementary material.

Supplementary Material 1

## Data Availability

All data supporting the findings of this study are available within the paper and its Supplementary Information.
