# Supplementary material for: Ultrasound and radiographic outcomes of the Coxaflex brace in the treatment of developmental dysplasia of the hip (DDH): a multicentre prospective study
Source: Eur J Orthop Surg Traumatol. 2026 Sep 10;36(1):375. doi: 10.1007/s00590-026-04942-4 (PMC13562303; doi:10.1007/s00590-026-04942-4)
Supplement: Supplementary file 1 [file 590_2026_4942_MOESM1_ESM.docx]

| **Parameter** | **Value** |
| --- | --- |
| Total hips evaluated | 44 |
| Unilateral cases | 22 |
| Bilateral cases | 11 |
| Right hips | 18 |
| Left hips | 26 |
| Female patients | 28 |
| Male patients | 5 |
| Median age at diagnosis (T0) | 46 ± 40 days |
| Age range at diagnosis | 4–150 days |
| Hips with positive Ortolani manoeuvre | 19 |
